# Subtle variation in size and shape of the whole forewing and the red band among co‐mimics revealed by geometric morphometric analysis in *Heliconius* butterflies

**DOI:** 10.1002/ece3.3916

**Published:** 2018-02-19

**Authors:** Dirleane O. Rossato, Danessa Boligon, Rodrigo Fornel, Marcus R. Kronforst, Gislene L. Gonçalves, Gilson R. P. Moreira

**Affiliations:** ^1^ Programa de Pós‐Graduação em Ecologia Instituto de Biociências Universidade Federal do Rio Grande do Sul Porto Alegre Brazil; ^2^ Programa de Pós‐Graduação em Ecologia Universidade Regional Integrada do Alto Uruguai e das Missões Erechim Brazil; ^3^ Department of Ecology and Evolution University of Chicago Chicago MI USA; ^4^ Programa de Pós‐Graduação em Biologia Animal Instituto de Biociências Universidade Federal do Rio Grande do Sul Porto Alegre Brazil; ^5^ Departamento de Recursos Ambientales Facultad de Ciencias Agronomicas Universidad de Tarapacá Arica Chile

**Keywords:** evolution, heliconian butterflies, mimetic rings, phenotypic variation, sexual selection

## Abstract

*Heliconius* are unpalatable butterflies that exhibit remarkable intra‐ and interspecific variation in wing color pattern, specifically warning coloration. Species that have converged on the same pattern are often clustered in Müllerian mimicry rings. Overall, wing color patterns are nearly identical among co‐mimics. However, fine‐scale differences exist, indicating that factors in addition to natural selection may underlie wing phenotype. Here, we investigate differences in shape and size of the forewing and the red band in the *Heliconius* postman mimicry ring (*H. erato phyllis* and the co‐mimics *H. besckei*,* H. melpomene burchelli, and H. melpomene nanna*) using a landmark‐based approach. If phenotypic evolution is driven entirely by predation pressure, we expect nonsignificant differences among co‐mimics in terms of wing shape. Also, a reinforcement of wing pattern (i.e., greater similarity) could occur when co‐mimics are in sympatry. We also examined variation in the red forewing band because this trait is critical for both mimicry and sexual communication. Morphometric results revealed significant but small differences among species, particularly in the shape of the forewing of co‐mimics. Although we did not observe greater similarity when co‐mimics were in sympatry, nearly identical patterns provided evidence of convergence for mimicry. In contrast, mimetic pairs could be distinguished based on the shape (but not the size) of the red band, suggesting an “advergence” process. In addition, sexual dimorphism in the red band shape (but not size) was found for all lineages. Thus, we infer that natural selection due to predation by birds might not be the only mechanism responsible for variation in color patterns, and sexual selection could be an important driver of wing phenotypic evolution in this mimicry ring.

## 1 INTRODUCTION

Neotropical *Heliconius* butterflies represent a conspicuously variable group from a morphological perspective (Holzinger & Holzinger, 1994), generally considered as a good model of evolutionary studies. Species exhibit wide intra‐ and interspecific variation in warningly colored wing patterns (Brown, 1979; Sheppard, Brown, Benson, & Singer, 1985). Groups of unpalatable species that have converged on the same warning pattern are considered as Müllerian mimicry “rings” (Mallet & Gilbert, 1995).

The adaptive nature of warning color patterns in *Heliconius* butterflies has been demonstrated experimentally in the wild by Benson (1972), Mallet and Barton (1989), and Kapan (2001). According to Joron (2009), mimicry is advantageous to mimetic butterflies, first by lessening the risk of predation by birds, which associate their warning color pattern with unpalatability. Second, because variant individuals existing in a given population may not be recognized as unpalatable, they are likely under greater predation risk. However, there is little information about the threshold for variation in the size and shape of these signals under natural conditions, if any (Mérot, Poul, Therv, & Joron, 2016), as well as the effect of concurrent selective pressures. The existence of phenotypic variation also may be advantageous to bird predators, because those individuals that better associate the unpalatability signal on the butterfly wing may have greater survivorship (Mallet & Barton, 1989). Learning in this case may be associated with several phenotypic, behavioral, and ecological factors associated with the butterflies, for example: (1) color (Svádová et al., 2009) and pattern (Ihalainen, Lindström, Mappes, & Puolakkainen, 2008) of the wings; (2) level of unpalatability (Ihalainen, 2006); and (3) relative frequency of the co‐mimics (Mérot et al., 2016; Rowland, Wiley, Ruxton, Mappes, & Speed, 2010; Speed, 2001). Variation in the former factors has not been quantitatively examined in the wild for any *Heliconius* mimicry ring. Unless predators are unable to detect fine‐scale differences, it is expected that natural selection alone would eventually lead to identical patterns of wing shape for each mimetic pair in *Heliconius* butterflies. In other words, “the ultimate prediction of Müllerian mimicry is that butterflies of a similar size should all ultimately converge on the same color pattern” (Brower, 1996; Mallet, Jiggins, & McMillan, 1996).

However, some degree of imperfection in mimicry is possible, especially if the cost to the mimetic butterfly for mate discrimination is greater than the benefit of mimicry protection. Phenotypic resemblance among co‐mimics could also impose costs due to possible mistakes in species identity during courtship (Estrada & Jiggins, 2008). Consequently, there could be a conflict between the outcomes of natural and sexual selection in mimetic species. Resemblance might also evolve under several selective pressures acting in a sex‐specific manner (Su, Lim, & Krushnamegh, 2015). Thus, additional, nonmutually exclusive, hypotheses may explain the existence of nonidentical mimics in the wild, including the “eye‐of‐the‐beholder,” where imperfect mimicry might be attributable to differences between the dimensions of organisms that humans notice versus the ones their ecologically relevant signal receivers pay attention to (Kikuchi & Pfennig, 2013). Similarly, the “mimetic breakdown” (Brower, 1960) assumes that imprecise mimicry reflects a trade‐off between gene flow and selection. Moreover, some wing traits could have evolved under concurrent pressures (see Rossato et al., 2018) with, for example, the shape of the red band being influenced by sexual selection in *Heliconius* (Emsley, 1970).

Finally, morphological similarities between Müllerian co‐mimics might be influenced not only by natural selection favoring accurate mimicry, but also by the genetic architecture underlying variation in wing phenotypes (Mérot et al., 2016), including introgression (e.g., *H. melpomene* and *H. timareta*; Heliconius Genome Consortium 2012; Pardo‐Diaz et al., 2012). Wing color pattern is controlled by few loci of major effect in *Heliconius erato* and *Heliconius melpomene* (Baxter et al., 2008). Thus, a common “tool kit” of genes, consisting of approximately five unlinked genetic loci that control almost all of the variation, has been used repeatedly by different species to produce both convergent and divergent wing patterns (Huber et al., 2015; Joron et al., 2006; Kronforst et al., 2006). Three of these loci that control size and shape of wing color patterns have been characterized at a molecular level: (1) the transcription factor *optix,* which controls the distribution of red color pattern across the wings (Reed et al., 2011), Kronforst & Papa, 2015), (2) the cell cycle regulator *cortex*, responsible for yellow patterning (Nadeau et al., 2016), and (3) *WntA*, a signaling ligand that controls melanin patterning across the wing (Martin et al., 2012).

**Figure 1:**
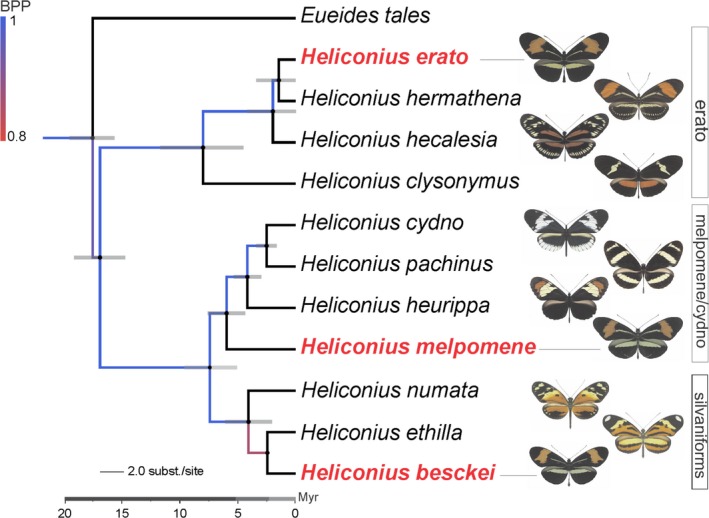
Phylogenetic tree of major clades within *Heliconius*. Illustrations on the right side depict wing pattern. The postman mimicry‐ring pattern is represented by the three species marked in red: *H. erato* (“erato” clade), *H. melpomene* (“melpomene/cydno” clade), and *H. besckei* (“silvaniform” clade). The tree was adapted from Kozak et al. (2015) including taxa of interest to this study

In this study, we evaluate fine‐scale morphological differences in the whole forewing and the red band trait among members of the postman mimicry ring of *Heliconius* butterflies. We explored the species *H. erato phyllis,* and its distantly related Müllerian co‐mimics *H. besckei*,* H. melpomene nanna,* and *H. melpomene burchelli* (Figure 1) that display the same warningly colored wing patterns in local populations, yet exhibit pattern diversity between geographic regions. Landmark and contour analysis based on semilandmarks were used to characterize spatial variation in forewing size and shape, and in the red band in *H. erato phyllis* across its distributional range, including areas of sympatry with *H. besckei, H. melpomene nanna,* and *H. melpomene burchelli*. *H. erato phyllis* is known to vary substantially in space, in terms of overall wing size and shape, due in part to its use of several species of host plants (passion vines) that also differ in their distribution (Jorge, Estrela, Klaczko, Moreira, & Freitas, 2011; Rodrigues & Moreira, 2002). However, there are no data about whether this wing pattern variation is spatially structured. There is also a lack of information about variation in size of the phenotypic signals that predators may use as cues, with the exception of those provided by Klein and Araújo (2013) for populations of *H. erato phyllis* and *H. besckei*.

If phenotypic resemblance among co‐mimics resulted mainly by predation pressure (i.e., convergence for mimicry; Kapan (2001); Mallet and Barton (1989)), we expected (1) nonsignificant differences among species (i.e., nearly identical co‐mimics) considering the size and shape of whole forewing and (2) reinforcement of wing patterning when co‐mimics are in sympatry. Alternatively, significant differences might exist in the size and shape of the red band, as this trait is also involved in sexual communication (Estrada & Jiggins, 2008), and therefore could be influenced by sexual selection. Finally, we inferred evolutionary patterns using the mitochondrial gene *Cytochrome oxidase I* (*CoI*) and the wing patterning gene *optix*, to evaluate evidence for introgression, which is also involved in morphological resemblance. We chose *optix*, in particular, because it is responsible for red wing pattern variation across *Heliconius* species (Reed et al., 2011).

## 2 MATERIALS AND METHODS

### 2.1 Species samples

**Figure 2:**
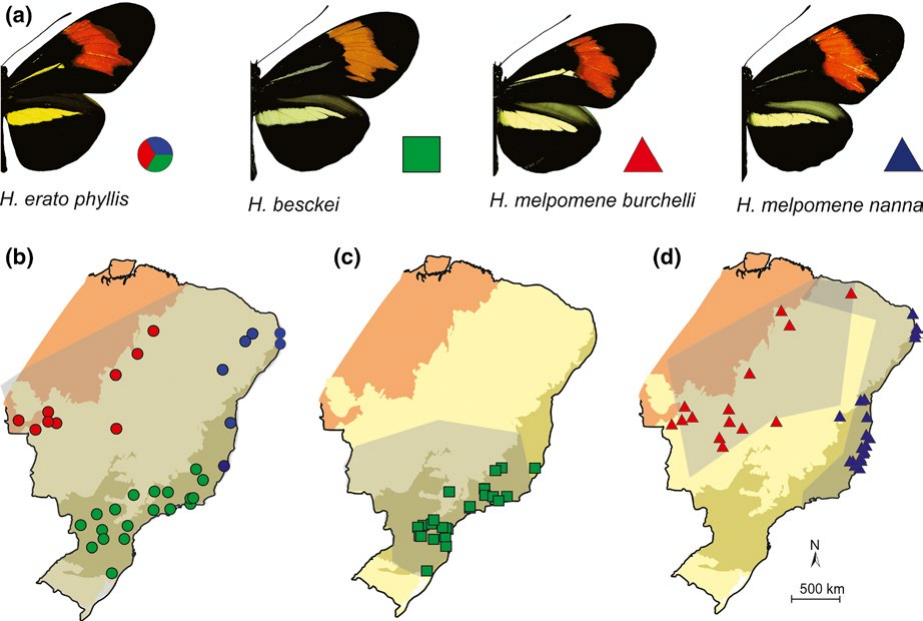
Distribution of species in the “postman” mimicry ring in Brazil. (a) Specimens from each co‐mimic of the mimicry ring. Maps represent the geographic locations of samples used in this study, as follows: (b) *H. erato phyllis*, with samples located in the central, northern, and southern regions, represented by red, blue, and green circles, respectively; (c) *H. besckei* (green squares); and (d) *H. melpomene burchelli* and *H. melpomene nanna* (red and blue triangles, respectively). Gray areas show the overall distribution of each mimetic‐ring member, according to Rosser et al. (2012). Biogeographical subregions are shown in brown (Amazon Forest), pale yellow (Chacoan), and pale green (Atlantic Rain Forest), following Morrone (2006). Photographs: GRP Moreira

Variation in overall forewing size and shape, and in the forewing red band, was analyzed in 229 field‐collected dried specimens of *H. erato phyllis*,* H. besckei*,* H. melpomene burchelli,* and *H. melpomene nanna* (Table 1). Specimens were chosen randomly, according to availability (mainly from the insect collection at the Universidade Federal do Paraná—UFPR, Brazil), until at least 20 specimens for each species or subspecies had been examined, following the geographic boundaries established by Brown (1979) and Rosser, Phillimore, Huertas, Willmott, and Mallet (2012) (but see Section 2.2 in Dell'Erba, Kaminski, and Moreira (2005) for distribution of *H. melpomene nanna*). In the case of *H. erato phyllis*, those that were distributed outside the geographic boundaries of the other members of the mimetic ring under study were excluded from the sample. Thus, for this taxon, the distribution of samples was drawn from three different regions in Brazil, forming a priori areas of sympatry with *H. melpomene burchelli*,* H. melpomene nanna,* and *H. besckei*, in central, northern, and southern Brazil, respectively (Figure 2, Table 1). The two subspecies of *H. melpomene* (*H. m. burchelli* and *H. m. nanna*) and *H. besckei* have smaller distributional ranges and are allopatric to each other.

**Table 1:** Individuals of *Heliconius* surveyed in the morphometrics analysis, including site(s) sampled (state: municipality), sympatric co‐mimic species, region (see Section 2 for details) and number of specimens separated by males and females

Species/subspecies	Site(s)^a^	Co‐mimic sympatric^b^	Region	Specimens	
				Male	Female
*Heliconius besckei* (Hb)	RS: São Francisco de Paula	Hep	Southern	2	0
	SC: Brusque, Iraputã, Joinville, Porto União and São Bento do Sul	Hep	Southern	4	1
	PR: Castro, Curitiba, Guarapuava, Morretes, Ponta Grossa, Prudentópolis, Paranagua, Tunas do Paraná	Hep	Southern	14	13
	SP: Bocaina, São Paulo	Hep	Southern	4	4
	RJ: Itatiaia, Mangaratiba, Petrópolis	Hep	Southern	2	4
	ES: Santa Teresa	Hep	Southern	1	2
	MG: Brumadinho, Caeté, Cambuquira and Carmo do Rio Claro	Hep	Southern	5	1
*Heliconius erato phyllis* (Hep)	RS: São Francisco de Paula	Hb	Southern	2	0
	SC: Nova Teutônia, São Bento do Sul	Hb	Southern	3	3
	PR: Cascavel, Curitiba, Fenix, Guarapuava, Jundiaí do Sul, Tunas do Paraná	Hb	Southern	5	3
	SP: São Paulo, Ubatuba, Bocaina	Hb	Southern	1	1
	RJ: Rio de Janeiro, Petrópolis, Teresópolis	Hb	Southern	3	3
	MG: Carangola, Cambuquira, Marliéria, Poços de Caldas, Caeté	Hb	Southern	2	2
	MT: Alto Xingu, Cáceres, Diamantino, Pontes e Lacerda, Chapada dos Guimarães, Jangada, Poconé	Hmb	Central	7	6
	GO: Ilha do Bananal, Goiás, Planaltina	Hmb	Central	2	3
	MA: Feira Nova do Maranhão	Hmb	Central	0	1
	CE: Ubajara	Hmb	Northern	2	4
	TO: Pedro Afonso	Hmb	Northern	1	0
	ES: Linhares, Sooretama	Hmn	Northern	0	1
	BA: Senhor do Bonfim, Camacan	Hmn	Northern	2	0
	PB: Santa Teresinha, Patos, João Pessoa	Hmn	Northern	2	3
	PE: Recife	Hmn	Northern	2	3
*Heliconius melpomene burchelli* (Hmb)	MT: Alto Araguaia, Alto Xingu, Barra do Garça, Cáceres, Chapada dos Guimarães, Diamantino, Nova Xavantina	Hep	Central	20	16
	MA: Feira Nova do Maranhão; Imperatriz	Hep	Central	12	4
	TO: Pedro Afonso	Hep	Central	0	3
	GO: Ilha do Bananal, Iporá, Mineiros, Planaltina	Hep	Central	1	1
	DF: Brasilia	Hep?	Central	1	0
	CE: Ubajara	Hep	Northern	1	1
*Heliconius melpomene nanna* (Hmn)	RN: Natal	Hep?	Northern	0	1
	PB: João Pessoa	Hep	Northern	1	3
	PE: Goiana, Recife, São Lourenço da Mata	Hep	Northern	1	3
	BA: Camacan, Itamaraju, Itamari, Jitaúna, Mucuri, Prado, São João do Paraíso	Hep	Northern	5	2
	ES: Baixo Guandu, Colatina, Conceição da Barra, Itapina, Jacaripe, Linhares, Pedro Canário, Santa Teresa, Sooretama	Hep	Northern	22	11
	MG: Aimorés	Hep?	Northern	2	1

a See Appendix S1 for site details.

b Question mark indicates that co‐mimics were not recorded in this study but potentially occurs in the area.

Field‐collected specimens (IBAMA/ICMBio license number 2024629) of *H. erato*,* H. besckei,* and *H. melpomene* stored at −20°C in the tissue collection of the Laboratório de Morfologia e Comportamento de Insetos (LMCI) of the Universidade Federal do Rio Grande do Sul (UFRGS), Brazil, were used for molecular phylogenetic analyses (*n* = 143 samples).

### 2.2 Morphometric data

**Figure 3:**
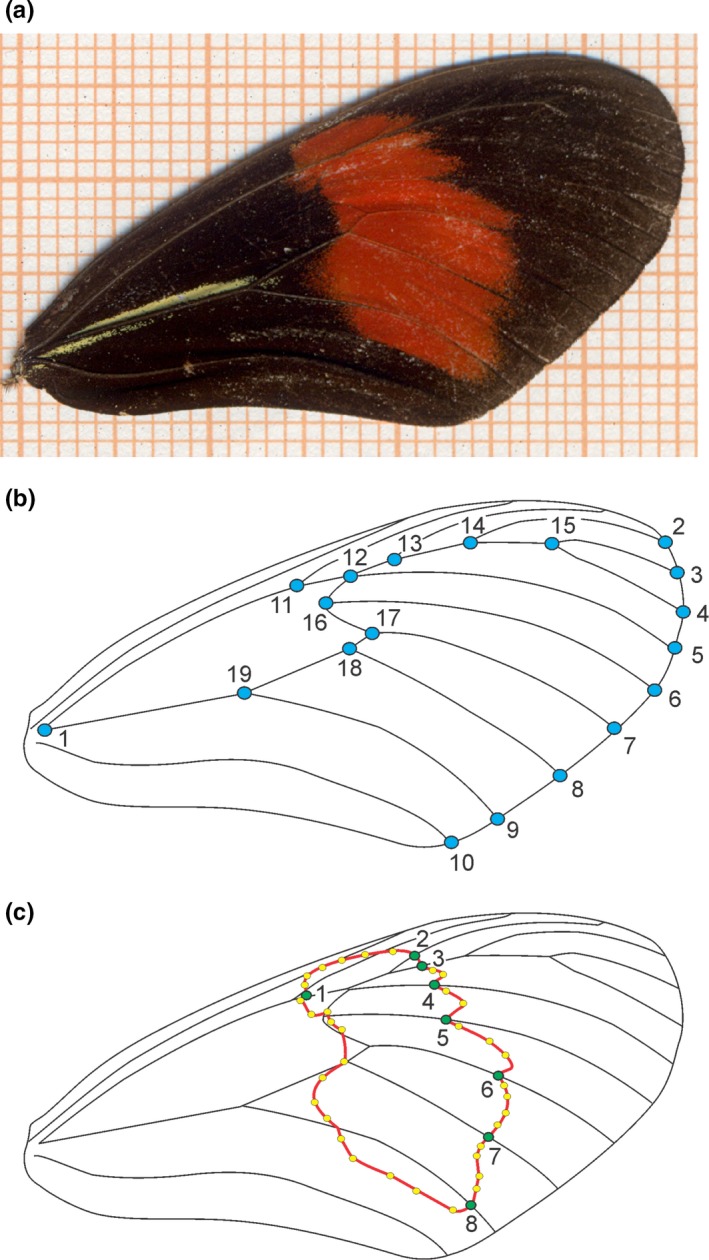
Location of landmarks on the forewing of *Heliconius melpomene burchelli*. (a) Photographed wing on millimeter paper. (b) Type I landmarks on whole wing, indicated by blue circles. (c) Type II landmarks (green circles) and semilandmarks (yellow circles) on red band

Dorsal surfaces of individual forewings were photographed by the same person (DOR) using a Sony Cybershot H20 digital camera, 5‐megapixel resolution, Iso200, one‐shot, flash off, and macro function activated (Figure 3a). The dorsal surface was chosen because it is likely to be subject to natural and sexual selection. In addition to the whole wing, we analyzed the size and shape of the red forewing band. Here, we focus on this wing color trait (and not the basal yellow stripe, for example) because it is known to be used as a visual cue in *H. erato phyllis* courtship and is likely also to be important for *H. melpomene* (Emsley, 1970). We used a total of 19 landmarks (Jorge et al., 2011) for the entire wing (Figure 3b) and eight landmarks plus 35 semilandmarks for the red band (Figure 3c) (for a complete description of the landmarks, see Table S1). Landmarks and semilandmarks were digitized by the same person (DOR), using TPSDig 2.17 (Rohlf, 2013).

Coordinates from the entire forewing and the red band were superimposed using a generalized Procrustes analysis (GPA) (Dryden & Mardia, 1998) using R Studio 0.97.551 (R Development Core Team, 2013) and Matlab 7.10.0.499 (Matlab & Statistics Toolbox Release, 2012). GPA removes differences unrelated to shapes, such as scale, position, and orientation (Adams, Rohlf, & Slice, 2004; Rohlf & Slice, 1990). To analyze the red band using semilandmarks, we created a slider file using TPSUtil 1.46 (Rohlf, 2010b). Points were adjusted by sliding along the outline curve until they matched as closely as possible the positions of the corresponding point on a reference specimen (Bookstein, 1997). The consensus configuration (mean shape) was calculated, and the difference among mean landmarks and individual landmarks resulted in a residual matrix (Jolliffe, 1986). This matrix was used in a principal components analysis (PCA) as the new shape variables. This procedure allowed us to reduce the dimensionality of the dataset and to work with independent variables (Cordeiro‐Estrela, Baylac, Denys, & Marinho‐Filho, 2006).

For analysis, we used R Studio and the libraries MASS (Venables & Ripley, 2002), ape 1.8‐2 (Paradis et al., 2006), and ade4 (Dray & Dufour, 2007). Geometric morphometrics procedures were carried out with RMORPH software: a geometric and multivariate morphometrics library for R (Baylac, 2008).

Comparative analyses of shape and size of the forewing and red band were used to evaluate evidence for geographic convergence between co‐mimics and differences between sexes. Samples were grouped in different ways for analyses focused on mimetic convergence versus sexual dimorphism. To infer the resemblance between co‐mimics, *H. erato phyllis* samples were clustered into three different geographic groups, as previously mentioned. These included 28, 19, and 13 individuals that occurred in sympatry with *H. besckei*,* H. melpomene burchelli,* and *H. melpomene nanna*, respectively (Figure 2). For comparison between sexes, we considered each species and subspecies separately, thus all geographic groups were grouped into one *H. erato phyllis* sample, resulting in four groups used to evaluate the existence of sexual dimorphism.

### 2.3 Forewing and red band size

Images were scaled using software IMP—CoordGen6f (Sheets, 2001), in order to compare size among members of the mimetic ring. Size was estimated as the log‐transformed centroid size, which represents the square root of the sum of squared distances of each landmark from the centroid of the configuration (Bookstein, 1991). We performed a one‐way analysis of variance (ANOVA), followed by Tukey's pairwise comparison tests, to determine whether forewing and red band size differed among mimicry‐ring members. ANOVA was also used to evaluate the effect of different pressures (mimicry convergence and sexual selection) on the whole forewing and red band in *H. erato phyllis*. Additionally, we performed Student's *t*‐tests to determine differences between males and females within the co‐mimic groups (*H. besckei, H. erato phyllis, H. melpomene burchelli,* and *H. melpomene nanna*). Regression analyses were performed between log‐centroid size of the whole forewing and red band to test for the existence of allometry, in relation to each member regarding isometry, and between genders within each mimicry‐ring member. Slope lines and intercepts were compared using one‐way analysis of covariance (ANCOVA). Allometric analyses and graphs were performed with GraphPad Prism 5.00 (Motulsky & Christopoulos, 2003) and edited in CorelDraw X4 (Corel Corporation).

### 2.4 Forewing and red band shape

Variation in the shape of the whole forewing and the red band was explored using principal components analyses. Analyses of shape variation, based on the first two principal components of the wing and red band, were conducted with TPSRelw 1.49 (Rohlf, 2010a) and edited in CorelDraw software. The consensus configurations for the whole forewing and the red band were obtained for each mimetic‐ring member. Then, we carried out a multivariate analysis of variance (MANOVA) of shape variables with the factors species/subspecies and sex. The shape discrimination for each co‐mimic and geographic groups of *H. erato phylis* was investigated using linear discriminant analysis (LDA), based on all PCs that explained 99% of the shape differences. Subsequently, we assessed the posterior probability of classification to each one, which depends on the relative distance of the specimens to the group means. We also calculated the Mahalanobis distance for the morphometric data from the whole forewing and the red band. With the corresponding Mahalanobis distances, we generated neighbor‐joining trees.

### 2.5 Evolutionary patterns

Resemblances between *Heliconius* co‐mimetic species could be partially driven by genetic similarities due to shared evolutionary history, particularly, those coding for the red band. Thus, we inferred gene trees for *CoI* and *optix*, using haplotypes observed in *H. erato phyllis*,* H. besckei,* and *H. melpomene*, to compare topologies in order to investigate whether these unlinked genes resulted in different evolutionary scenarios. The evolutionary patterns were also used to infer whether resemblance resulted from introgression of red wing patterning among species in the postman mimicry ring. We performed a Bayesian analysis with Yule prior on branching rates, which were allowed to vary under a relaxed clock model with an uncorrelated log‐normal distribution (Drummond, Ho, Phillips, & Rambaut, 2006), using the GTR model. The genetic divergence between pairs of taxa was used to compare the whole forewing and red band shape distances with calculated values using *p*‐distance, with 1000 bootstrap replications.

Molecular data were obtained from fresh‐collected samples of *Heliconius* species. High‐quality DNA was purified from larval tissue using cetyl trimethyl ammonium bromide (CTAB). DNA amplicons were amplified using polymerase chain reaction (PCR) for sections of two genes: (1) 821 base pairs (bp) of the mitochondrial gene *cytochrome c oxidase* subunit *I* (*CoI*) and (2) 782 bp of *optix*. *optix* was chosen because it controls red patterning in *Heliconius* (Reed et al., 2011). Primers and PCR conditions used were as described by Beltrán, Jiggins, Brower, Bermingham, and Mallet (2007) and Hines et al. (2011) for *CoI* and *optix*, respectively. Aliquots of PCR products were treated with Exonuclease I and FastAP Thermosensitive Alkaline Phosphatase (Thermo Scientific), sequenced using the BigDye chemistry, and analyzed on an ABI3730XL (Applied Biosystems Inc.). Sequences were aligned and visually inspected using Clustal X implemented in MEGA v6 (Tamura, Stecher, Peterson, Filipski, & Kumar, 2016) running in full mode with no manual adjustment. All sequences have been submitted to NCBI GenBank under the accession numbers: *CoI/optix*:* H. besckei* KJ468656‐64/KM099363‐67, *H. erato phyllis* KJ468620‐55/KM099342‐61, and *H. melpomene* KJ468665‐70/KM099368‐73.

To evaluate the influence of phylogenetic constraints on the phenotypic resemblance, two matrices were built using the four taxa (*H. erato phyllis*,* H. besckei*,* H. melpomene burchelli*, and *H. melpomene nanna*): (1) pairwise genetic distance calculated based on Tamura–Nei substitution model using concatenated sequences (*CoI* and *optix*) and (2) Mahalanobis distance based on the whole forewing and the red band. Positive association (*r* > 0) between the whole forewing and red band distances was inferred through a Mantel test using Pearson's correlation coefficient in the software XLSTAT (Addinsoft).

## 3 RESULTS

### 3.1 Variation in the size of whole forewing and red band among co‐mimics

**Figure 4:**
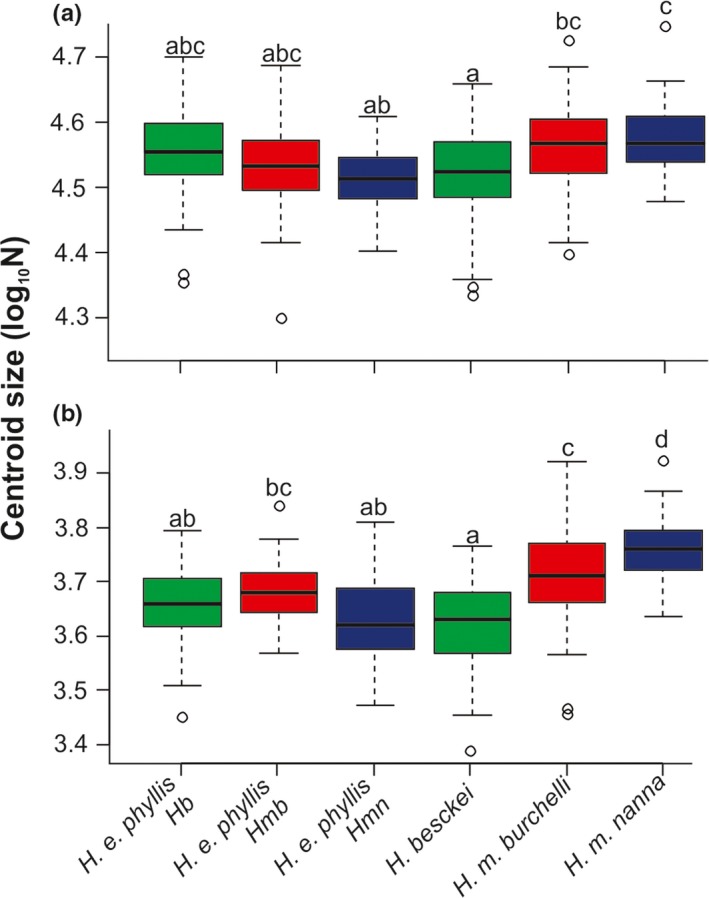
Variation in size of forewings of *Heliconius* mimicry‐ring members and geographic groups of *H. erato phyllis*. (a) Whole wing. (b) Red band. Box plots followed by the same letter do not differ statistically (Student′s *t*‐tests, α = 0.05). Groups of *H. erato phyllis* compared to each co‐mimic are represented by the same color; *H. e. phyllis* Hb, sympatric with *H. beskei*;* H. e. phyllis* Hmb, with *H. melpomene burchelli;* and *H. e. phyllis* Hmn with *H. melpomene nanna*

The ANOVA based on whole forewing centroid size showed significant differences among groups (*F*_5,223_ = 5.777, *r* ^2^ = .09, *p *<* *.001). There was no difference in the forewing size between the three populations of *H. erato phyllis* (*p *>* *.70), nor comparing groups of *H. erato phyllis* to *H. beskei* (*p *=* *.598), to *H. melpomene burchelli* (*p *=* *.055), and the allopatric groups to *H. melpomene nanna*. However, *H. erato phyllis* had a smaller forewing size than *H. melpomene nanna*, when in sympatry (Tukey′s HSD test; Figure 4a). The ANOVA based on red band centroid size also showed significant differences (*F*_5,223_ = 22.45, *r* ^2^ = .32, *p *<* *.001). The corresponding pairwise Tukey's HSD tests were all significant (*p *<* *.001) for species level, and the results were similar to the forewing size. However, the size of red band in the three groups of *H. erato phyllis* did not differ from each other, both in allopatry and sympatry with co‐mimics; but, when in sympatry with *H. melpomene nanna,* the size of the red band was smaller. In fact, the red band from *H. melpomene nana* was bigger than all others co‐mimics (Figure 4b).

### 3.2 Variation in the shape of whole forewing and red band among co‐mimics

**Figure 5:**
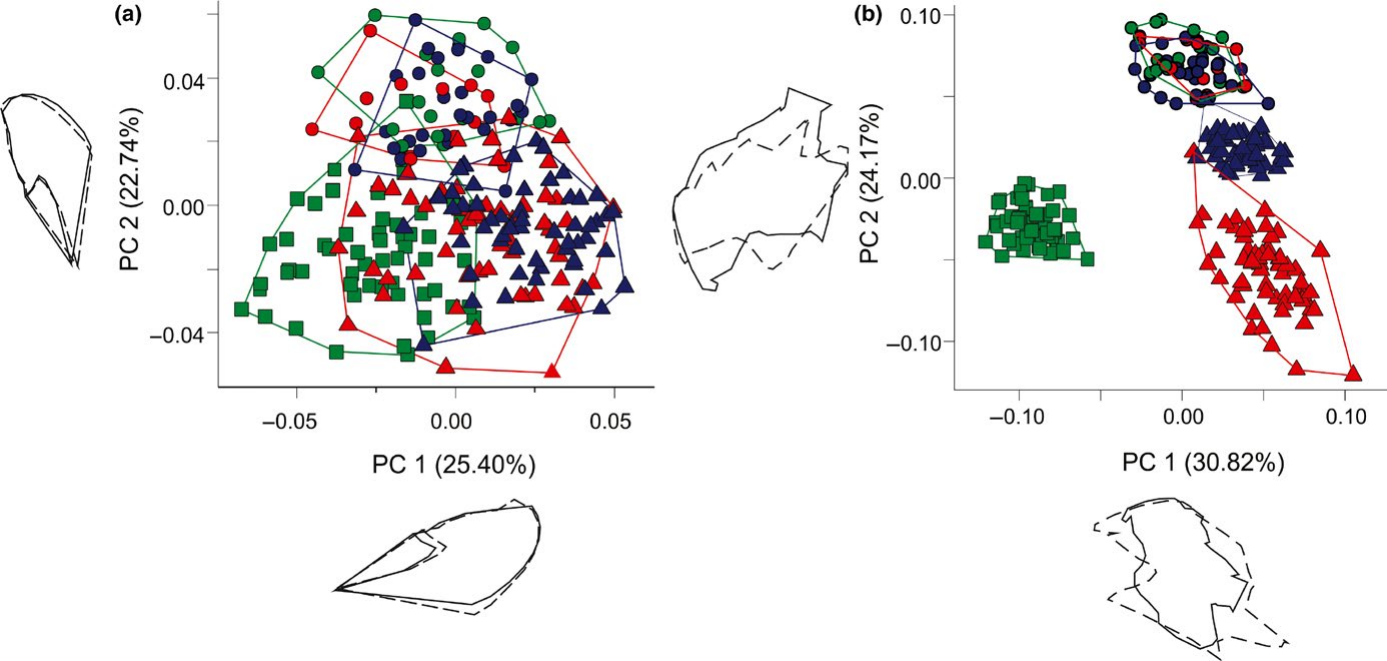
First two axes of the principal components analysis (PCA) on shape residuals for the whole forewing (a) and red band (b) of *Heliconius* mimicry‐ring members. Percentage of shape variation explained by each PCA is shown in parentheses. The shape deformations are shown near each axis, where the solid line represents the shape at minimum values and the dashed line represents the shape at maximum values. Circles represent *H erato phyllis* individuals in sympatry with: green, *H. besckei*; red, *H. melpomene burchelli*; and blue, *H. melpomene nanna*. The green squares and red and blue triangles represent, respectively, *H. besckei*,* H. melpomene burchelli,* and *H. melpomene nanna*

**Figure 6:**
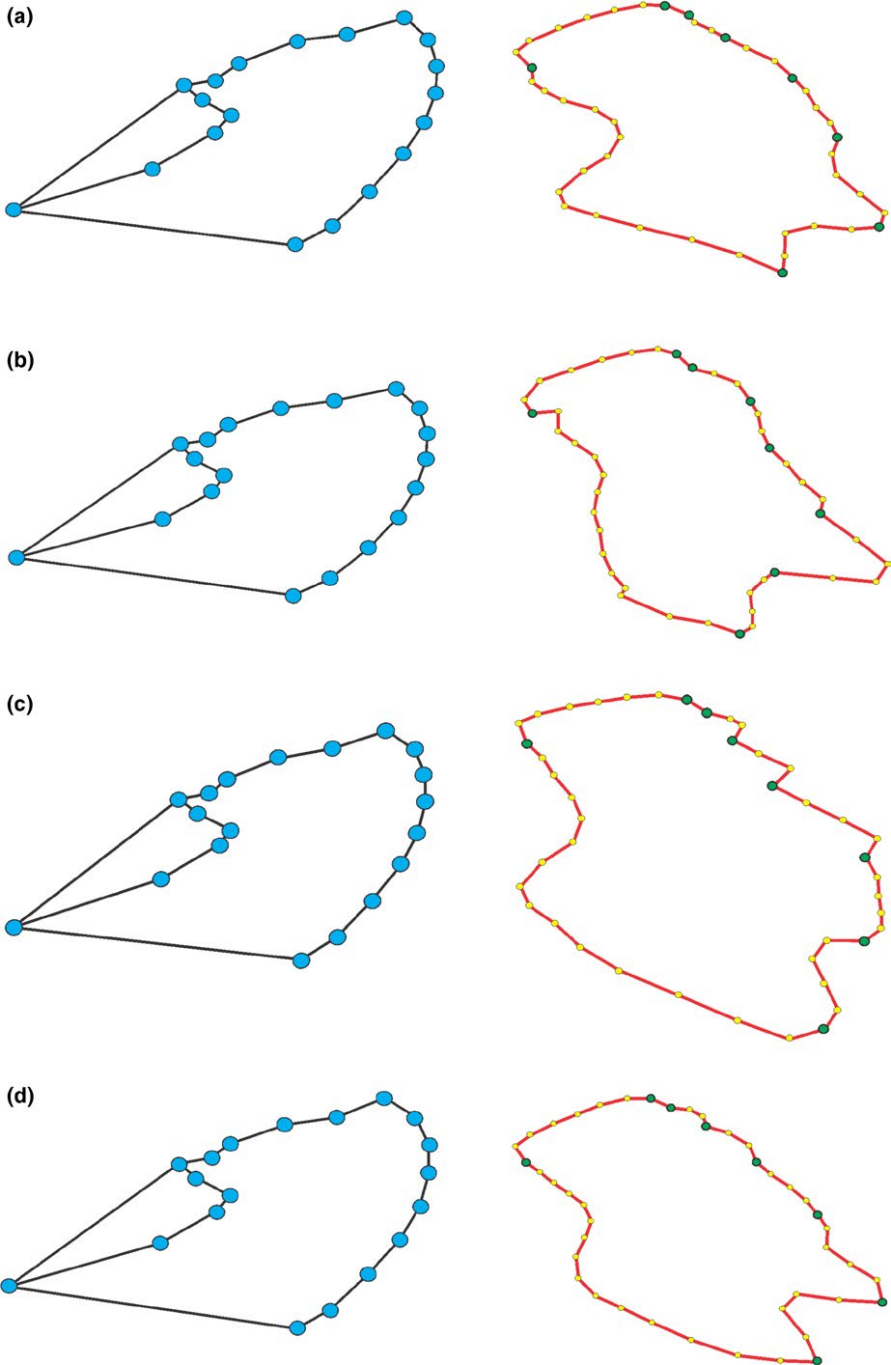
Consensus configuration (mean shape) for the whole forewing (left column) and red band (right column) of *Heliconius* mimicry‐ring members. (a) *H. erato phyllis*. (b) *H. besckei*. (c) *H. melpomene burchelli*. (d) *H. melpomene nanna*. Landmark types I and II, and semilandmarks are indicated by blue, green, and yellow circles, respectively

The results of the MANOVA based on shape of the forewing and red band showed significant differences among all members of the mimicry ring, between sexes (discussed in details below), and also for the interaction between these two factors (Table 2). The first ten PCs for the whole wing shape explained 85.12% of the variation. There was no clear separation among mimicry‐ring members in this case for the first two PCs that together explained ~48% of variation (Figure 5a). The consensus of forewing shape for each species and subspecies is shown in Figure 6. Thus, although the MANOVA test indicated statistically significant difference, the differences in the shape of the whole forewing were relatively small. The posterior probability of classification to each co‐mimic species and subspecies, using 34 PCs, was 100% for each species and 83.33% and 94.23% for the subspecies of *H. melpomene*,* H. melpomene burchelli,* and *H. melpomene nanna,* respectively. The posterior probability of classification to the geographic groups of *H. erato phyllis* was 78.57%, 84.21%, and 83.33% for those sympatric with *H. besckei*,* H. melpomene burchelli,* and *H. melpomene nanna,* respectively. For the red band, the first ten PCs explained 81.44% of the shape variation, and the first two PCs accounted for more than 50%. In this case, there was a clear separation of all mimicry‐ring members, but not within *H. erato phyllis* (Figure 5b). In the first PC, *H. besckei* was separated from the other species, while the second PC separated *H. erato phyllis* from *H. melpomene nanna*, and *H. melpomene burchelli* from *H. besckei* with partial overlap on this axis. The greater variation for the red band within a given mimicry ring, when compared to the whole forewing, is seen in the corresponding consensus shape (Figure 6). The posterior probability of classification to each co‐mimic, based on the red band, using 37 PCs, was 100% for each co‐mimic species and subspecies, and 78.57%, 73.68, and 38.46% for the geographic groups of *H. erato phyllis* sympatric with *H. besckei*,* H. melpomene burchelli,* and *H. melpomene nanna,* respectively.

**Table 2:** MANOVA results for shape variation of the forewing and red band in *Heliconius* mimicry‐ring members, taking into account as separate groups the two subspecies of *H. melpomene* and the three geographic areas for *H. erato phyllis* (total = six groups, 229 individuals) and sex

Trait	Category	λ Wilks	*F*	*p*
Whole wing	Sex	0.408	7.11	<.001*
	Groups	0.008	7.98	<.001*
	Sex × Groups	0.284	1.41	.001*
Red band	Sex	0.506	4.78	<.001*
	Groups	<0.001	22.29	<.001*
	Sex × Groups	0.116	2.65	<.001*

a Significant *p*‐value α = 0.05.

### 3.3 Effect of sex and geographic groups on forewing in Heliconius mimetic‐ring members

The ANOVA results comparing the effect of sex and geographic group on red band size suggest that both mimicry convergence and sexual dimorphism act in *H. erato phyllis* (*F*_5,54_ = 2.60, *r* ^2^ = .12, *p *=* *.03) and that there is an interaction between these factors. The red band was smallest in the *H. erato phyllis* group that was sympatric with *H. melpomene nanna*. We did not find significant difference in whole forewing size (*F*_5,54_ = 0.82, *r* ^2^ = −.01, *p *=* *.54). The MANOVA results for shape variation in *H. erato phyllis* indicate that both selective factors affect these wing traits, but they act on different structures (Table 3). For instance, while geographic group was important to forewing shape, the red band shape varied according to sex.

**Table 3:** MANOVA results for shape variation of the forewing and red band in *Heliconiu erato phyllis*, taking into account geographic group and sex (total =* *three groups, 60 individuals, 29 female and 31 male)

Trait	Category	λ Wilks	*F*	*p*
Whole wing	Sex	0.179297	2.2268	.036*
	Groups	0.016563	3.2935	<.001*
	Sex × Groups	0.076047	1.2776	.211
Red band	Sex	0.150821	2.73911	.013*
	Groups	0.220248	0.55012	.985
	Sex × Groups	0.052858	1.62951	.054

a Significant *p*‐value, α = 0.05.

### 3.4 Differences between males and females

**Figure 7:**
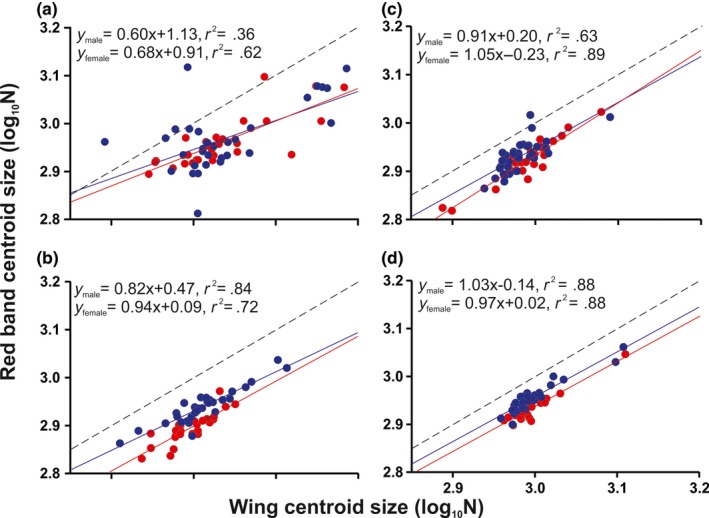
Variation in size of the red band in relation to forewing size among *Heliconius* mimicry‐ring members. (a) *H. erato phyllis*. (b) *H. besckei*. (c) *H. melpomene burchelli*. (d) *H. melpomene nanna*. Males and females are represented by blue and red circles, respectively. Isometry is indicated by dashed line

**Figure 8:**
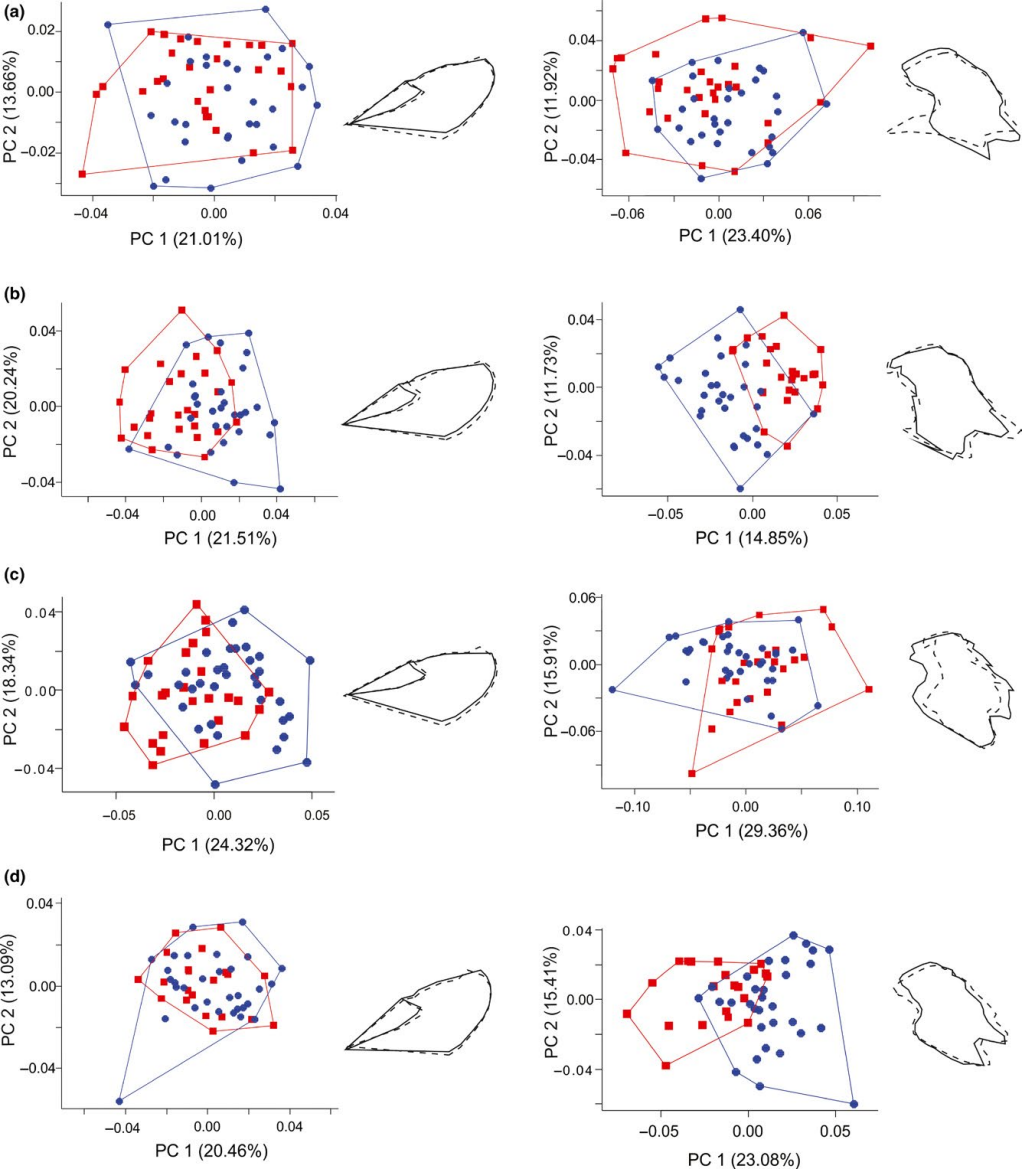
First two axes of the principal components analysis (PCA) on shape residuals for the whole forewing (left column) and red band (right column) of *Heliconius* mimicry‐ring members, comparing males (blue circles) and females (red circles). (a) *H. erato phyllis*. (b) *H. besckei*. (c) *H. melpomene burchelli*. (d) *H. melpomene nanna*. Percentage of shape variation explained by each PCA is shown in parentheses. The shape deformations are shown next to each graph, where the dashed line represents the shape at minimum values and the solid line represents the shape at maximum values

There was no significant difference between males and females in forewing size for all species and subspecies (Table 4). However, the red band size showed a significant difference between sexes for all species and subspecies, being relatively bigger in males in relation to females (Figure 7), except *H. erato phyllis*, in which no difference was found (Table 4). The size relationship between the wing and the red band showed negative allometry only in *H. erato phyllis* (*y* = 0.64*x* + 1.03, *r* ^2^ = .45, *p *<* *.0001, Figure 7a). For the other mimicry‐ring members, the relationship was isometric, with the corresponding linear regression equations as follows: *H. besckei* (*y* = 0.94*x* + 0.99, *r* ^2^ = .71, *p *=* *.48, Figure 7b), *H. melpomene burchelli* (*y* = 0.98*x* + 0.01, *r* ^2^ = .75, *p *=* *.75, Figure 7c), and *H. melpomene nanna* (*y* = 0.98*x* + 0.006, *r* ^2^ = .781, *p *=* *.80, Figure 7d). For all mimicry‐ring members, males and females did not differ from each other in terms of allometric coefficient. However, comparing them to the isometric line, males and females of *H. e. phyllis* and males of *H. besckei* showed negative allometry. For the *H. melpomene* subspecies, males and females did not differ from isometry (Table 5; Figure 7). The MANOVAs on the whole forewing and red band shape for males and females showed significant differences for all mimicry‐ring members (Table 6). In general, the main differences between sexes were concentrated in the first PC, and therefore, we show only the shape variation for PC1 in the PCA (Figure 8).

**Table 4:** Effect of sex on size of whole forewing and on size of red band in *Heliconius* mimicry‐ring members

Species	Whole wing			Red band		
	*t*	*df*	*p*	*t*	*df*	*p*
*H. erato phyllis*	0.46	57.18	.65	0.30	52.82	.77
*H. besckei*	0.76	52.34	.45	−4.10	47.80	.0002^a^
*H. melpomene burchelli*	0.39	32.86	.70	−2.00	40.40	.05^a^
*H. melpomene nanna*	0.39	48.31	.70	−3.22	50.28	.002^a^

a Significant value for Student′s *t*‐tests, α = 0.05.

**Table 5:** Comparison of allometric coefficients in wing versus red band centroid size (regression analysis) within and between sexes in *Heliconius* mimicry‐ring members

Species	Sex	*n*	Slope			Intercept		
			β	*F*	*p*	α	*F*	*p*
*H. erato phyllis*	Male versus female^a^			0.14	.71		0.24	.63
	Male^b^	31	0.60	7.06	.010*	1.13	c	c
	Female^b^	29	0.68	10.02	.002*	0.91	c	c
*H. besckei*	Male versus female			0.80	.37		51.80	<.0001
	Male	32	0.82	7.39	.01*	0.47	c	c
	Female	25	0.94	0.27	.61	0.09	918.55	<.0001*
*H. melpomene burchelli*	Male versus female			0.97	.33		9.40	.003*
	Male	34	0.95	0.12	.73	0.11	164.81	<.0001*
	Female	24	1.09	1.03	.31	−0.33	378.01	<.0001*
*H. melpomene nanna*	Male versus female			0.35	.56		48.69	<.0001*
	Male	32	0.94	0.99	.32	0.14	470.41	<.0001*
	Female	22	0.94	0.86	.36	0.12	724.32	<.0001*

a Comparison of allometric coefficients between males and females;

b Comparison of allometric coefficients in relation to their isometric line;

c Slope differs so much, that it is not possible to test whether the intercept differs significantly;

*Significant *p*‐value, α = 0.05.

**Table 6:** MANOVA results for effect of sex on forewing shape in *Heliconius* mimicry‐ring members

	Species/subspecies	λ Wilks	*F*	*p*
Whole wing	*H. besckei*	0.093	4.98	<.001*
	*H. erato phyllis*	0.158	3.17	.003*
	*H. melpomene burchelli*	0.213	2.19	.027*
	*H. melpomene nanna*	0.139	2.35	.044*
Red band	*H. besckei*	0.134	4.18	<.001*
	*H. erato phyllis*	0.153	2.63	.013*
	*H. melpomene burchelli*	0.227	2.50	.010*
	*H. melpomene nanna*	0.877	9.399	<.001*

a Significant *p*‐value, α = 0.05.

### 3.5 *Optix* and COI phylogeny and genetic distances

**Figure 9:**
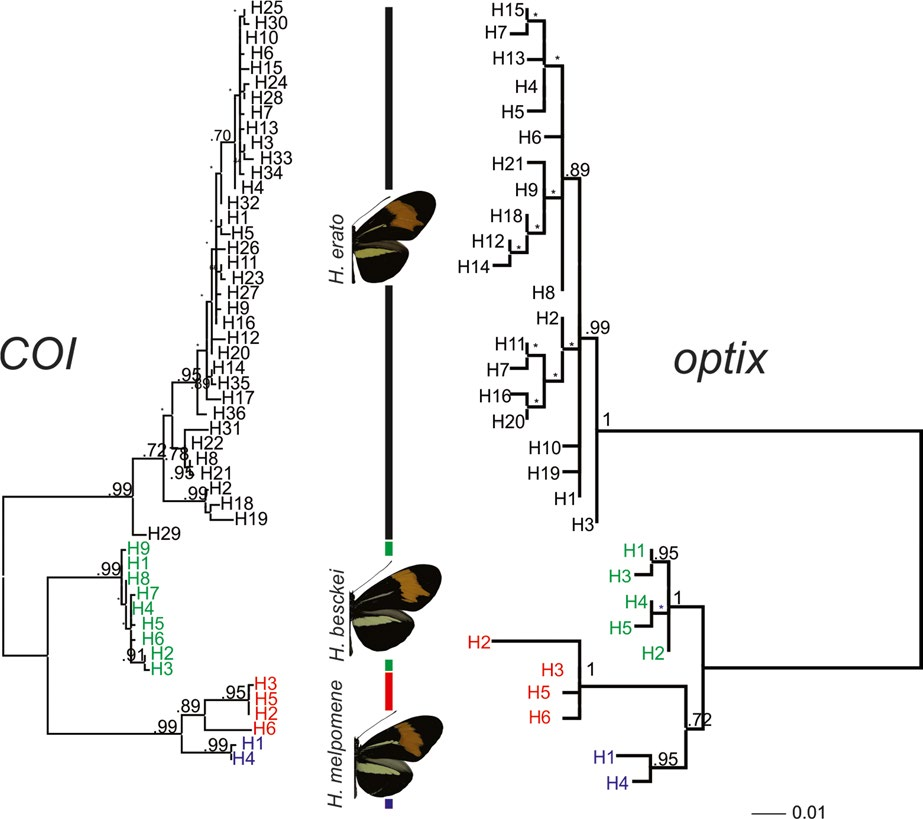
Evolutionary relationships among haplotypes of the postman mimicry ring members (*H. erato phyllis, H. besckei, H. melpomene burchelli*, and *H. melpomene nanna*) from *cytochrome oxidase c subunit I* [*CoI*] and *optix* gene sequences. Numbers above branches indicate Bayesian posterior probability support

**Figure 10:**
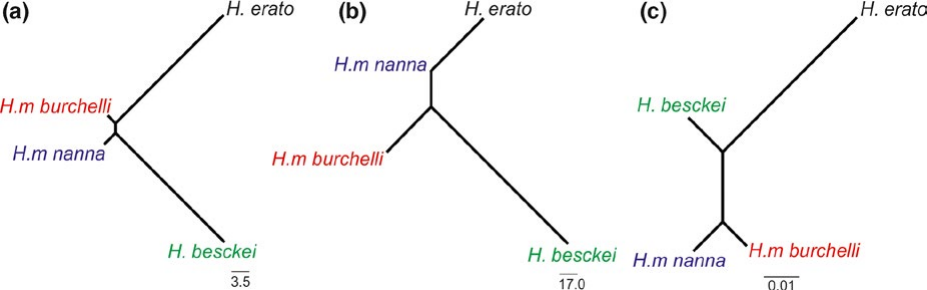
Distance among postman mimicry ring members (*H. erato phyllis, H. besckei, H. melpomene burchelli, H. melpomene nanna*). Mahalanobis distances for shape on (a) the whole forewing and (b) red band; and (c) genetic distances based on Tamura–Nei parameter using concatenated loci (*cytochrome oxidase c* subunit *I *+ *optix*)

We sequenced a 1.6‐kb region of *CoI* and *optix* to infer phylogenetic relationships among *H. erato phyllis* and the co‐mimics and identify potential patterns of introgression. Gene trees reconstructed based on *CoI* and *optix* resulted in similar topologies (*H. erato phyllis* (*H. besckei and H. melpomene*)), but with subtle differences in branch lengths (Figure 9). Thus, no obvious discordance was evident, although *H. besckei* has previously been shown to have acquired wing patterning mimicry, and the genomic segment around *optix*, from *H. melpomene* via introgression (Zhang, Dasmahapatra, Mallet, Moreira, & Kronforst, 2016). On the other hand, neighbor‐joining (NJ) trees of Mahalanobis distance based on the whole wing and the red band indicated that *H. erato phyllis* was more similar to *H. melpomene* subspecies (Figure 10). Mantel tests indicated that genetic distance was positively correlated with morphology (Mahalanobis) distance for both the whole forewing (*r* = .90; *p *=* *.20) and red band (*r* = −.99; *p *=* *.92) shapes, although these were not statistically significant.

## 4 DISCUSSION

### 4.1 Convergence and “advergence” in wing pattern among co‐mimics

The morphological diversity of animals is generally assumed to be adaptive and shaped by natural selection. Nevertheless, traits can be influenced by multiple selective pressures, some of which may act in conflicting directions (Petrović et al., 2015). By refining the morphology of wing traits, we found that natural and sexual selection might be acting in opposing directions (as firstly pointed by Estrada and Jiggins (2008)) in members of the postman mimicry ring. Our results demonstrate that variation in size and shape is quite distinct between co‐mimics considering the whole forewing and the red band.

First, the lack of differentiation in size and shape of whole forewing indicates that co‐mimics are nearly identical, that is, they likely converged for mimicry as a response to predation pressure. This is particularly clear for the shape of whole forewing of the two broadly codistributed species, *H. erato phylis* and the subspecies of *H. melpomene* (Figure 10), which are phenotypically similar, despite being more distantly related. The evolutionary history of these species should be taken into account to explain such resemblance (Brown, Sheppard, & Turner, 1974; Eltringham, 1916; Flanagan et al., 2004; Quek et al., 2010; Turner & Mallet, 1996). However, the color pattern similarity observed between *H. erato* and *H. melpomene* might also result from an interaction between environmental changes, host plant distribution, and phenotypic plasticity (Rossato et al., 2018). Additionally, a phylogenetic constraint could not be ruled out as we found a relevant signal for correlation between the wing pattern and genetic distance, although this was not statistically significant due to the low sample size. Convergence was also observed for the size of whole forewing considering geographic groups of *H. erato phyllis* sympatric with each co‐mimic, except *H. melpomene nanna*. Overall, the strong cohesion of *H. erato phyllis* samples from different geographic groups suggests great maintenance in the shape of the forewing and red band, compared with *H. melpomene nanna*. This could be important to address the competition observed with *H. erato phyllis*, which might have resulted in the use of different species of host plants (passion vines, *Passiflora* spp.) by *H. melpomene nanna* in Northeast Brazil. In this region, *H. melpomene* shows similar phenotypic plasticity to *H. erato phyllis*, in which the wing size and shape depend on passion vines that also differ in distribution (Jorge et al., 2011; Rodrigues & Moreira, 2002).

Second, our results revealed that differences in the shape of the red band were diagnosable and consistent among members of the postman mimicry ring, including the subspecies of co‐mimics. The correct classification, based on shape, of each species (100%) or subspecies (>80%) showed the dissimilarity between geographic group of *H. erato phyllis* and sympatric co‐mimics. Accordingly, co‐mimics are not identical in the way that humans view their shape.

Thus, natural selection due to predation by birds may be only partly responsible for shaping forewing traits. It is possible that when the butterflies are actively flying, predators cue on the red band on a broader scale and do not recognize these phenotypic variants as different entities in the wild. Co‐mimics may not be identical because they display a more generalized signal in which subtle differences are not perceived or are ignored by predators (Rowe, Lindström, & Lyytinen, 2004). Additionally, nonidentical convergence does not mean absence of mimicry, but could be result of other selective pressures (Srygley, 1994). In the MANOVA results (Table 2), the differences in shape are related to the interaction between sex and groups, suggesting that sexes and geographic groups result in different shape (Table 2). Co‐mimetic species may, in fact, benefit from the presence of other co‐mimics, even when these are slightly dissimilar (Rowe et al., 2004; Ihalainen, Lindström, & Mappes, 2007; Rowland, Ihalainen, Lindström, Mappes, & Speed, 2007; Merót et al., 2016; Finkbeiner, Briscoe, & Mullen, 2017). In the area of Brazil where we conducted this study, there are other members of the postman mimicry ring that occur at low densities, for example, *Eresia lansdorfi* (Nymphalidae). While this species displays a red forewing band similar to others in the postman mimicry ring, it may be a Batesian mimic and was not considered a Müllerian co‐mimic. At least initially during the learning phase, chemical differences among co‐mimics may be more important than visual similarities (Darst & Cumming, 2006; Lindström, Alatalo, & Mappes, 1997). *Heliconius* butterflies have conspicuous exocrine glands on the last abdominal segments, which were originally presumed to be associated with defense in both sexes (Müller, 1912; Ross et al., 2001), but have not been explored in detail in the context of Müllerian mimicry. Lately, these structures have been associated with the production (males) and storage and dispersal (females) of antiaphrodisiacs (Gilbert, 1976; Schulz, Estrada, Yildizhan, Boppré, & Gilbert, 2008). However, these functions may be not mutually exclusive. The possibility that chemicals produced by these structures have a role in species recognition also remains to be explored.

Studies that evaluate the role of different selective pressures on mimicry should pay attention to the potential impact of selection acting on different aspects of the wing (Rossato et al., 2018). While the size of the whole forewing does not seem to be affected by sexual or natural selection, the size of red band of *H. erato phyllis* males differed when in sympatry with each subspecies of *H. melpomene*. This result, and the great difference between *H.erato phyllis* sympatric with *H. melpomene,* suggests some selective pressure could act differently in this geographic site. Moreover, this was not observed when we evaluated separately co‐mimics and sex in *H. erato phyllis*, suggesting that the size depends on the interaction between group and sex. Additionally, different factors could act on the evolution of the forewing shape, such as differences in flight (Cespedes, Penz, & DeVries, 2015) and abiotic factors such as host plant usage (Jorge et al., 2011). The spatial distribution of each plant could vary substantially among areas, which could result in several phenotypically distinct co‐mimics, defined as races or subspecies according to host plant occurrence (Brown, 1979; Hines et al., 2011; Rosser et al., 2012; Turner, 1981).

### 4.2 Species identity and sexual dimorphism

We found evidence for the existence of forewing variation between all co‐mimics (including the subspecies of *H. melpomene*) and between males and females of each species. Differences between the subspecies cannot be explained only by mimicry with *H. erato*. Thus, the differences in shape are consistent to each co‐mimic (high percent of the correct classification in the same species). The differences in shape are bigger considering the red band of each co‐mimic, suggesting a maintenance of this trait for sexual communication. It is also possible that they are used in courtship, because the MANOVA results demonstrate the red band shape is strongly influenced by sex (Table 4). As sexual dimorphism in the shape of the red band was found in all lineages explored in this study, the results also suggest that sexual selection may be involved in the evolution of this trait. The use of color and wing shape as visual stimuli during courtship in both sexes of *H. erato* was first demonstrated by Crane (1955). According to Emsley (1970), the red color is in fact used as a visual cue in *H. erato phyllis* courtship and is likely also to be important for *H. melpomene melpomene*. However, preliminary tests conducted by Emsley suggested that the yellow color present on the hind wings of the mimicry‐ring members, and which was not taken into account here, is important for *H. besckei*. It is unclear whether such subtle differences in shape could be efficiently used as visual cues.

Similarly to Ramos and Freitas (1999), Jorge et al. (2011), and Klein and Araújo (2013), we found no sexual dimorphism in overall forewing size in *H. erato phyllis*. Analogous results were obtained for *H. besckei* and the two subspecies of *H. melpomene*. However, when the size of the red band was taken into account, all members showed sexual dimorphism, except *H. erato phyllis*. Klein and Araújo (2013) found similar results for the latter species and also for *H. besckei*, suggesting that in these species, this trait might be under sexual selection pressure. In general, both sexes are choosy in *Heliconius* and our results support the idea that wing patterns might be important cues for male and female mate choice. Although difficult to specifically test, this might explain why it is not just one sex that deviates from completely convergent mimicry.

The negative allometry found for the forewing red band in *H. erato phyllis* was interpreted as evidence for the existence of a size threshold as an effective visual cue used in the context of either predation and/or courtship. In other words, with individuals varying up to 50% in size in the wild (Rodrigues & Moreira, 2002), those with small forewings compensate by having a proportionally larger red band. This appears not be the case for *H. melpomene nanna*, perhaps because on average, they vary less and are naturally larger than *H. erato phyllis*.

Finally, the evolutionary relationships revealed by phylogenies inferred from *CoI* and *optix* were identical, consistent with the known phylogeny and a history of introgression between *H. besckei* and *H. melpomene*. Probably, distinct alleles are involved in wing color shape and the type of variation that we observed in the postman mimicry ring is either controlled by a different allele or region than that studied. Moreover, epistatic interaction between *optix* and the modifier locus *N* results in a narrow forewing red band (Martin et al., 2014), pointing to a more complex genetic architecture underlying the red wing pattern.

## 5 CONCLUSION

In this study, we demonstrated the existence of distinct patterns of variation in shape and size of the whole forewing and the red band among co‐mimics of the *Heliconius* postman ring, which suggests mimicry convergence and sexual selection acting in opposing directions. The two most widely distributed species, and distantly related, *H. erato phyllis* and *H. melpomene,* are the most similar regarding whole forewing and red band. Also, we found consistent differences in the red band that enables us to distinguish among co‐mimics, including subspecies, even when in sympatry. Sexual dimorphism not in size but in shape was found in relation to the red band suggesting that sexual selection might play a role in the evolution of this trait. Thus, we inferred that natural selection due to predation by birds, which in theory would lead to nearly identical color patterns, is not the only mechanism responsible for the variation in these phenotypic patterns. Whether the mimicry‐ring members tested here use these differences as visual cues to identify, compete, and/or choose their partners remains to be tested. As postulated by Mallet et al. (1996), mimicry theory is still open to discussion, and “poses more questions than it answers.”

## AUTHOR CONTRIBUTIONS

GRPM coordinated the collection of samples and directed the research. DOR, DB, and RF analyzed and interpreted the geometric morphometric dataset. DOR, MK, and GLG collected the molecular data. DOR and GLG analyzed the molecular dataset and oversaw manuscript preparation. All authors contributed to the project design and in preparing the manuscript.

## CONFLICT OF INTEREST

None declared.
