# Supplementary material for: Subtle variation in size and shape of the whole forewing and the red band among co‐mimics revealed by geometric morphometric analysis in Heliconius butterflies
Source: Ecol Evol. 2018 Feb 19;8(6):3280–95. doi: 10.1002/ece3.3916 (PMC5869215; doi:10.1002/ece3.3916)
Supplement: Supplementary file 1 [file ECE3-8-3280-s001.docx]

**Table S1**. Morphological definition of forewing landmark and semi-landmark depicted in Figure 3.

|  | Mark Anatomic | Type | Description |
| --- | --- | --- | --- |
| Forewing | 1 | Land I | Humeral vein (hv) basis |
|  | 2 | Land I | Radial 3 (R3) distal point |
|  | 3 | Land I | Radial 4 (R4) distal point |
|  | 4 | Land I | Radial 5 (R5) distal point |
|  | 5 | Land I | Media 1 (M1) distal point |
|  | 6 | Land I | Media 2 (M2) distal point |
|  | 7 | Land I | Media 3 (M3) distal point |
|  | 8 | Land I | Cubital anterior 1 (CuA1) distal point |
|  | 9 | Land I | Cubital anterior 2 (CuA2) distal point |
|  | 10 | Land I | Anal 1 (1A) distal point |
|  | 11 | Land I | Radial 1 (R1) proximal point |
|  | 12 | Land I | Media 1 (M1) proximal point |
|  | 13 | Land I | Radial 2 (R2) proximal point |
|  | 14 | Land I | Radial 3 (R3) proximal point |
|  | 15 | Land I | Radial 4-5 (R4 and R5) proximal point |
|  | 16 | Land I | Media 2 (M2) proximal point |
|  | 17 | Land I | Media 3 (M3) proximal point |
|  | 18 | Land I | Cubital anterior 1 (CuA1) proximal point |
|  | 19 | Land I | Cubital anterior 2 A (CuA2) proximal point |
| Red band | 1 | Land II | Radial 1 (R1) |
|  | 2 | Land II | Radial 2 (R2) |
|  | 3 | Land II | Radial 3 (R3) |
|  | 4 | Land II | Media 1 (M1) |
|  | 5 | Land II | Media 2 (M2) |
|  | 6 | Land II | Média 3 (M3) |
|  | 7 | Land II | Cubital anterior 1 (CuA1) |
|  | 8 | Land II | Cubital anterior 2 (CuA2) |
|  | 9 to 14 | Semi | between landmark 1 and 2 |
|  | 15 to 16 | Semi | between landmark 3 and 4 |
|  | 17 to 18 | Semi | between landmark 4 and 5 |
|  | 19 to 21 | Semi | between landmark 5 and 6 |
|  | 22 to 25 | Semi | between landmark 6 and 7 |
|  | 26 to 29 | Semi | between landmark 7 and 8 |
|  | 30 to 43 | Semi | between landmark 8 and 1 |

Land I = Landmark type I, Land II = Landmark type II, Semi = Semi landmark.
