# Supplementary material for: Subtle variation in size and shape of the whole forewing and the red band among co‐mimics revealed by geometric morphometric analysis in Heliconius butterflies
Source: Ecol Evol. 2018 Feb 19;8(6):3280–95. doi: 10.1002/ece3.3916 (PMC5869215; doi:10.1002/ece3.3916)
Supplement: Supplementary file 2 [file ECE3-8-3280-s002.docx]

**Appendix S1. *Heliconius* specimens used in morphometric and genetic analyses, listed per local and institution.**

Specimens were obtained from the following collections: DZUP, Coleção Entomológica Pe. Jesus Moure, Universidade Federal do Paraná, Paraná, Brazil; LMCI, Coleção de Tecidos, Laboratório de Morfologia e Comportamento de Insetos, Universidade Federal do Rio Grande do Sul, Rio Grande do Sul, Brazil. ɸ photographed geometric morphometric, Ψ used molecular analysis (Ψ^1^ = COI, Ψ^2^ = optix).

**a) *Heliconius besckei***

**Espirito Santo**:

Santa Teresa, 1.IX,1966, C. & C. T. Elias leg., 1♀ (DZUP ɸ) (Hb17);

Santa Teresa, 3.VII.1967, C. & C. T. Elias leg., 1♀ (DZUP ɸ) (Hb18);

Santa Teresa (R. Lombardia) 1.VIII.1966, Mielke, Elias & Brown leg., 1♂ (DZUP ɸ) (Hb116);

**Minas Gerais**:

Brumadinho, 19.XI.2009, G. R. P. Moreira leg., 1♀ (LMCI 90-01 ɸ Ψ^1^), 1♂ (LMCI 90-02 ɸ Ψ^1^);

Caeté (Gongo Soco), 20.XI.2009, G. R. P. Moreira leg., 9♂ (LMCI 88-01 ɸ Ψ^1^, LMCI 88-02 ɸ Ψ^1^, LMCI 88-03 Ψ^1^, LMCI 88-04 Ψ^1^, LMCI 88-05 Ψ^12^, LMCI 88-06 Ψ^1^, LMCI 88-07 Ψ^1^, LMCI 89-01 Ψ^1^, LMCI 89-02 Ψ^1^).

Cambuquira, 7.II.1985, Mielke & Casagrande leg., 1♀ (DZUP ɸ) (Hb21);

Carmo do Rio Claro, VIII.1948, Candido & ‘?’ col., 1♂ (DZUP, Coll. D’Almeida 6645, ɸ) (Hb102);

**Paraná**:

Castro, 15.XI.1971, Mielke & ‘?’ leg., 1♀ (DZUP ɸ) (Hb15);

Curitiba (Cascatinha), 8.V.1966, Mielke O leg., 2♀♀, 1♂ (DZUP ɸ) (Hb08) (Hb14) (Hb126);

Curitiba (Cascatinha) 19.V.1966, Mielke O leg., 1♀ (DZUP ɸ) (Hb09);

Curitiba (Cascatinha), 30.XI.1966, Mielke O leg., 1♀ (DZUP ɸ) (Hb16);

Curitiba (Cascatinha), 29.I.1967, Mielke O leg., 3♂♂ (DZUP ɸ) (Hb 119) (Hb 120) (Hb 121);

Curitiba (900m), 2.III.1968, Mielke O leg., 1♂ (DZUP ɸ) (Hb122);

Curitiba (Cascatinha), 1.XII.1967, Mielke O leg., 2♂♂ (DZUP ɸ) (Hb 123) (Hb 124);

Curitiba (Cascatinha) 9.X.1966, Mielke O leg., 1♂ (DZUP ɸ) (Hb125);

Curitiba, Colégio Politécnico, 31.III.2009, O. Mielke leg., 1♀ (LMCI 53-14 Ψ^1^)

Guarapuava (1000m), 28.I.1972, Mielke & Schneider leg., 1♀ (DZUP ɸ) (Hb07);

Guarapuava (1200m), 3.II.1976, Mielke & Buzzi leg., 1♂ (DZUP ɸ) (Hb110);

Morretes (Marumbi), 13.V.1967, Mielke & Laroca leg., 1♀ (DZUP ɸ) (Hb04);

Morretes (Marumbi), 19.V.1966, Bianeli S leg., 1♂ (DZUP ɸ) (Hb115);

Ponta Grossa, III.1958, 1♀ (DZUP, Coll F. Justus, ɸ) (Hb05);

Ponta Grossa (Lageado), III.1958,1♀ (DZUP , Coll F. Justus Jor, ɸ) (Hb06);

Ponta Grossa, III.1958, 1♂ (DZUP, Coll F. Justus, ɸ) (Hb118);

Ponta Grossa, VI.1941, 1♀ (DZUP , Coll F. Justus Jor, ɸ) (Hb10);

Ponta Grossa (Vila Velha, 1000m), 5.II.1967, Mielke leg., 1♀ (DZUP ɸ) (Hb11);

Ponta Grossa (Piriquitos, 1000m), 21.IV.1967, Moura & Mielke leg. 1♂ (DZUP ɸ) (Hb112);

Prudentópolis (1200 m), 2.II.1976, Mielke & Buzzi leg., 1♀ (DZUP ɸ) (Hb12);

Alexandra (Paranagua), 3.IV.1970, Lauterjung M leg., 1♀ (DZUP ɸ) (Hb13);

Alexandra (Paranagua), 6.IX.1968, Mielke O leg., 2♂♂ (DZUP ɸ) (Hb111) (Hb117);

Tunas do Paraná, 15.IV. 2008, Moreira GRP leg., 6♂ (LMCI 22-05 Ψ^1^, LMCI 52-20 Ψ^1^, LMCI 52-21 Ψ^1^, LMCI 52-24 Ψ^12^, LMCI 52-25 Ψ^12^, LMCI 52-26 Ψ^1^)

**Rio de Janeiro**:

Itatiaia (900m), 4.II.1936, 1♀ (DZUP, Coll. Gagarin, ɸ) (Hb23);

Itatiaia (800m), 26.II.1964, H. Ebert leg., 1♀ (DZUP, Coll. H. Ebert 434, ɸ) (Hb24);

Itatiaia (1400m), 20.XII.1957, 1♀ (DZUP, Coll. H. Ebert 828, ɸ), (Hb25);

Mangaratiba (Sahy), 4.V.1932, 1♂ (DZUP, Coll. Ferr. D’Almeida 6651, ɸ) (Hb108);

Petrópolis (Independência, 900m), 18.X.1939, 1♀ (DZUP, Coll. Gagarin, ɸ), (Hb22);

**Rio Grande do Sul:**

São Francisco de Paula, FLONA, 03.IV.2010, Millan C leg., 11♂ (LMCI 123-01 ɸ, LMCI 123-02 ɸ Ψ^1^, LMCI 24-11 Ψ^1^, LMCI 24-12 Ψ^1^, LMCI 24-13 Ψ^1^, LMCI 24-14 Ψ^1^, LMCI 27-08 Ψ^1^, LMCI 27-09 Ψ^1^, LMCI 27-10 Ψ^1^, LMCI 27-11 Ψ^1^, LMCI 123-3 Ψ^1^)

**Santa Catarina**:

Brusque, 27-30.III.1970, Becker CF leg., 1♂ (DZUP ɸ) (Hb114);

Iraputã, 2.III.1937, 1♂ (DZUP, Coll. D’Almeida 6644, ɸ) (Hb101);

Joinville, 25.III.1978, Mielke O leg., 1♂ (DZUP ɸ) (Hb109);

Joinville, 9.X. 1971, Mielke O leg., 1♂ (DZUP ɸ) (Hb113);

Porto União, 1♀ (DZUP, Coll. D’Almeida 6647, ɸ) (Hb02);

São Bento do Sul, II.2010, Moreira GRP leg., 6♂ (LMCI 110-82 Ψ^12^, LMCI 110-83 Ψ^1^, LMCI 110-84 Ψ^1^, LMCI 110-85 Ψ^12^, LMCI 110-87 Ψ^1^), 1♀ (LMCI 110-86 Ψ^1^);

**São Paulo**:

Bocaina (Bananal), 8.I.1937, Travassos Col., 1♂ (DZUP, Coll. D’Almeida 6650, ɸ) (Hb105);

São Paulo (Eldorado Represa Nova), 27.IV.1941, 1♀ (DZUP, Coll. D’Almeida 6643, ɸ) (Hb01);

São Paulo (capital), 3.VIII.1940, 1♀ (DZUP, Coll. D’Almeida 6646, ɸ) (Hb03);

São Paulo (Túnel da Mata Fria, 800m), 8.II.1985, Mielke & Casagrande leg., 1♀ (DZUP ɸ) (Hb20);

São Paulo (Ypiranga), VII.1941, 1♀ (DZUP, Coll. D’Almeida 6642, ɸ) (Hb26);

São Paulo (Cantareira), III.1934, L. Trav F., 1♂ (DZUP, Coll. D’Almeida 6653, ɸ) (Hb103);

São Paulo (Ipiranga), 19.VIII.1943, 1♂ (DZUP, Coll. D’Almeida 6652, ɸ) (Hb104);

b) *Heliconius erato phyllis*:

**Bahia**:

Senhor do Bonfim , 21.VI.1974, S. Laroca leg., 1♂ (DZUP ɸ) (Hep106)

Camacan (Serra Bonita), 17-23.II.2013, Moreira, G. R. M. leg., 1♂ (LMCI 209-27 ɸ Ψ^12^);

**Ceará:**

Ubajara, 2♂ (LMCI 183-02 Ψ^1^, LMCI 183-05 Ψ^1^), 4♀ (LMCI 183-01 Ψ^1^, LMCI 183-03 Ψ^12^, LMCI 183-04 Ψ^1^, LMCI 183-07 Ψ^1^)

**Espirito Santo**:

Linhares, V.1978, C. Elias leg., 1♀ (DZUP ɸ) (Hep04);

Sooretama, ReBio, 28.II. -2.III.2010, GRPM et al leg., 6♂ (LMCI 105-35 Ψ^2^, LMCI 105-36 Ψ^1^, LMCI 105-37 Ψ^1^, LMCI 105-38 Ψ^1^, LMCI 105-40 Ψ^2^, LMCI 105-41 Ψ^2^, LMCI 105-66 Ψ^1^, LMCI 105-67 Ψ^1^, LMCI 105-68 Ψ^1^’), 3♀ (LMCI 105-39 Ψ^1^, LMCI 105-40 Ψ^1^, LMCI 105-41 Ψ^1^)

**Goiás**:

Ilha do Bananal, 23.IX.1980, Gifford leg., 1♀, 1♂ (DZUP, Coll. Gifford, ɸ) (Hep01) (Hep 102);

Goiás, XI.1976, Gifford D. leg., 1♀ (DZUP, Coll. Gifford, ɸ) (Hep09);

Goiás, VII.1976, Grifford D. leg., 1♂ (DZUP, Coll Gifford, ɸ) (Hep 107);

Planaltina, 2♂ (LMCI 224-06 Ψ^2^, LMCI 224-10 Ψ^1^), 1♀ (LMCI 224-01 Ψ^12^)

**Maranhão**:

Feira Nova do Maranhão (Faz. Forquilha dos Brejos), 14-21.VIII.2011, Mielke O leg., 1♀ (DZUP ɸ) (Hep19);

**Mato Grosso**:

Alto Xingu, 18.VII.1978, Gifford leg., 2♀♀ (DZUP ɸ) (Hep02) (Hep03);

Alto Xingu, 18.VII.1978, Gifford leg., 1♂ (DZUP ‘Coll. Gifford.’, ɸ) (Hep104);

Cáceres, 19.XI.1984, Buzzi, Mielke, Elias, Casagrande leg., , 1♀ (DZUP ‘Proj. POLONOROESTE’ ɸ) (Hep06);

Cáceres, 16.XI.1984, Buzzi, Mielke, Elias, Casagrande leg. , 1♀ (DZUP ‘Proj. POLONOROESTE’ ɸ) (Hep08);

Diamantino (Faz. S. João), 21.XI.1984, Mielke & Casagrande leg, 1♀ (DZUP ɸ) (Hep 07)

Diamantino (Faz. S. João, Rio Arinos, 300-400m), 1-8.VIII.1974, H & HD Ebert leg., 2♂♂ (DZUP, Coll. H. Ebert, ɸ), (Hep116) (Hep 117)

Pontes e Lacerda (Fazenda Ouro Preto). 26.VII.2008, Queiroz & Silva leg, 1♂ (DZUP ɸ), (Hep101);

Chapada dos Guimarães (Buriti), 700m, 8.VIII.1973, 1♂ (DZUP, Coll. H. Ebert 658, ɸ), (Hep114);

Chapada dos Guimarães (Buriti, 700m), 23.XII.1968, 1♂ (DZUP, Coll H. Ebert 509, ɸ), (Hep115);

Jangada, 03.VI.2013, Rossato, DO leg., 1♀ (LMCI 226-04 ɸ Ψ^1^), 3♂ (LMCI 226-01 Ψ^1^, LMCI 226-02 Ψ^1,^ LMCI 226-03 ɸ Ψ^1^);

Poconé, 04.VI.2013, Rossato, DO leg., 1♀ (LMCI 227-01 Ψ^12^)

**Minas Gerais**:

Carangola (Fervedouro, 650m), 18.II.1972, Ebert, 1♀ (DZUP, Coll. H. Ebert 954, ɸ) (Hep17);

Cambuquira (900m), 19.IX.1969, H. Ebert leg., 1♀ (DZUP, Coll. H. Ebert 508, ɸ) (Hep21);

Marliéria (Parque Estadual do Rio Doce), 20.IX.1974, Gifford leg., 1♂ (DZUP ɸ) (Hep108);

Poços de Caldas (100m), 22.IV.1967, 1♂ (DZUP, Coll. H. Ebert 322, ɸ) (Hep120);

Caeté, Gongo Soco, 20.XI.2009, G. R. P. Moreira leg., 7♂ (LMCI 43-10 Ψ^1^, LMCI 43-12 Ψ^1^, LMCI 43-13 Ψ^1^, LMCI 43-14 Ψ^2^, LMCI 43-15 Ψ^1^, LMCI 43-16 Ψ^1^, LMCI 43-17 Ψ^1^, LMCI 43-19 Ψ^1^), 1♀ (LMCI 43-14 Ψ^1^).

**Paraíba**:

Santa Teresinha, 05.VIII.2011, Kerpel S leg., 1♀ (LMCI 5180-02 ɸ), 1♂ (LMCI 180-06 ɸ);

Patos, 27.VIII.2011, Kerpel S & Ferreira A leg., 1♀ (LMCI 181-02 ɸ), 1♂ (LMCI 181.01 ɸ);

João Pessoa, 14.XII.1952, Kesselring J leg., 1♂ (DZUP, Coll. Ebert 324, ɸ) (Hep 118);

**Paraná:**

Cascavel, 8.IX.1985, Mielke & Casagrande leg., 1♂ (DZUP ɸ), (Hep105);

Curitiba (Contro Politécnico), 31.IV.2009, K. Barão leg., 2♀ (LMCI 54-16 ɸ Ψ^1^, LMCI 54-25 Ψ^1^), 5♂ (LMCI 54-18 ɸ, LMCI 54-21 Ψ^1^, LMCI 54-23 Ψ^12^, LMCI 54-24 Ψ^1^, LMCI 54-25 Ψ^2^).

Fenix (300m), 23.XI.1986, Mielke & Casagrande leg., 1♂ (DZUP ‘PROFAUPAR’ ɸ), (Hep113);

Guarapuava (Santa Clara, 650m), 21.XI.1986, Mielke & Casagrande leg., 1♂ (DZUP ‘PROFAUPAR’ ɸ) (Hep112);

Jundiaí do Sul, 24.XI.1986, Mielke & Casagrande leg. , 1♀ (DZUP ‘PROFAUPAR’ ɸ), (Hep16);

Tunas do Paraná, 01.IV.2009, Moreira GRP leg., 1♀ (LMCI 52-60 ɸ Ψ^12^), 1♂ (LMCI 52-59 ɸ Ψ^12^);

**Pernambuco**:

Recife, 7.VII.1958, Ebert leg., 1♀ (DZUP, Coll. Ebert 327, ɸ) (Hep15)

Recife, 23.VIII.1958, Ebert leg., 1♀ (DZUP, Coll. Ebert 321, ɸ) (Hep22)

Recife (Camaragibe), 1.IV.1961, H. Ebert, 1♂ (DZUP, Coll. H. Ebert, ɸ) (Hep122)

Recife (Camaragibe), 27.IV.1958, 1♂ (DZUP, Coll. H. Ebert 328, ɸ) (Hep123)

**Rio de Janeiro**:

Rio de Janeiro (Lagoinha, 250m), 9.II.1952, H. Ebert 1♀ (DZUP, Coll. H. Ebert 86, ɸ), (Hep05);

Rio de Janeiro, 15.V.1941, 1♀ (DZUP, Coll. Gagarin, ɸ), (Hep14);

Petrópolis, 6.XII.1961, Gagarin leg., 1♂ (DZUP ɸ), (Hep103);

Petrópolis (Independência), 21.II.1934, Garfarin leg., 1♂ (DZUP, Coll. Gagarin, ɸ), (Hep111);

Teresópolis (Serra dos Orgãos, 25m), 20.12.1955, Ebert H. leg., 1♂ (DZUP, Coll. H. Ebert 87, ɸ) (Hep124);

**Rio Grande do Sul**:

São Francisco de Paula (FLONA), 20.IV.2006, Moreira GRP leg., 7♂ (LMCI 24-17 Ψ^1^, LMCI 24-18 Ψ^1^, LMCI 24-19 Ψ^12^, LMCI 27-01 Ψ^1^, LMCI 27-02 ɸ Ψ^1^, LMCI 27-03 ɸ, LMCI 27-04 Ψ^12^, LMCI 27-05 Ψ^1^);

**Santa Catarina**:

Nova Teutonia (300-500m), V.1981, Plaumann F leg., 1♀, 1♂ (DZUP ɸ) (Hep11) (Hep109);

Nova Teutonia (300-500m), II.1981, Plaumann F leg., 1♀, 1♂ (DZUP ɸ) (Hep13) (Hep110);

São Bento do Sul, II.2010, Moreira GRP leg., 3♀ (LMCI 110-154 ɸ Ψ^12^, LMCI 110-155 Ψ^1^, 110-156 Ψ^12^), 4♂ (LMCI 110-157 ɸ Ψ^12^, LMCI 110-158 Ψ^12^, LMCI 110-159 Ψ^1^, LMCI 110-161 Ψ^1^),

**São Paulo**:

São Paulo, 29.IV.1971, Mielke leg., 1♀ (DZUP ɸ) (Hep10);

Ubatuba (80 m), 27.10.1962, 1♀ (DZUP, Coll. Ebert 329, ɸ) (Hep18);

Bocaina, 5.III.1966, H. Ebert leg. 1♂ (DZUP, Coll. H Ebert 311, ɸ) (Hep119);

**Tocantins**:

Pedro Afonso, IX.1962, ‘?’ leg., 1♀ (DZUP, Coll H. Ebert, ɸ) (Hep20)

c) *Heliconius melpomene burchelli*

**Mato Grosso:**

Alto Araguaia, 11.VIII.1924, Ebert leg., 1♀ (DZUP, Coll. H. Ebert 566, ɸ) (Hmb23);

Alto Xingu (Porto Jacaré), VIII.1948, Candido J leg., 7♂♂, 2♀♀ (DZUP Coll.D´Almeida 6654-6662, ɸ), (Hbm01, 02, 101, 102, 103, 104, 105, 108, 109);

Barra do Garça (São Felix), 25.VII.1968, Claudionor Elias leg., 1♀, 8 ♂♂ (DZUP ɸ), (Hbm05) (Hmb122) (Hmb123) (Hmb125) (Hmb126) (Hmb127) (Hmb130) (Hmb131) (Hmb132);

Barra do Garça (Vale dos Sonhos, 400m), 22.VI.1972, Mielke & Brown leg., 3♂♂ (DZUP ɸ) (Hmb113) (Hmb124) (Hmb128);

Barra do Garça (, 520m), Mielke & Brown leg., 1♂ (DZUP ɸ), (Hmb133);

Cáceres, 19.XI.1984, Buzzi, Mielke, Elias & Casagrande leg., 1♀ (DZUP Proj. ‘Polonoroeste’ɸ), (Hmb14);

Chapada dos Guimarães (Buriti, 600m), 25.VI.1972, Mielke & Brown leg., 1♀ (DZUP ɸ) (Hbm04);

Chapada dos Guimarães (Buriti, 600m), 26.VI.1972, Mielke & Brown leg., 2♀♀, 1♂ (DZUP ɸ), (Hmb06) (Hmb07) (Hmb129);

Chapada dos Guimarães (Buriti, 700m), 22.XII.1968, 1♀ (DZUP, Coll. H. Ebert 494, ɸ) (Hmb18);

Chapada dos Guimarães, 23.XI.1983, Buzzi, ‘?’, Yamamoto & Hoffmann, 1♀ (DZUP ɸ) (Hmb22);

Chapada dos Guimarães, 28.III-8.IV.1972, C. Elias leg., 2♀♀ (DZUP Proj. ‘Polonoroeste’ ɸ), (Hmb16) (Hmb17);

Diamantino (Rio Arinos, Faz. S. João, 300-400m), 23.VII.1975, H. & H. D. Ebert leg., 1♀ (DZUP, Coll. H. Ebert 647, ɸ), (Hmb19);

Nova Xavantina, 14.I.1977, Gifford leg., 1♀ (DZUP, Coll. Gifford, ɸ), (Hmb15);

Nova Xavantina (Bacaba, UNIMAT), 17-19.VIII.1997, Mielke leg, 1♀ (DZUP ɸ), (Hmb20);

Nova Xavantina, 09.I.1977, Gifford leg., 1♀ (DZUP, Coll. Gifford, ɸ), (Hmb21);

**Goias**:

Ilha do Bananal, 23.IX.1980, Gifford leg., 3♀♀ (DZUP, Coll. Gifford, ɸ), (Hbm10) (Hmb11) (Hmb12);

Iporá (22Km oeste, 420m), 21.VI.1972, Mielke & Brown leg., 1♂ (DZUP ɸ), (Hmb111);

Mineiros (Parque das Emas), XI.1980, Gifford leg., 1♀ (DZUP, Coll. Gifford, ɸ), (Hbm13);

Planaltina, 2♂ (LMCI 224-22 Ψ^1^, LMCI 224-23 Ψ^1^);

**Distrito Federal:**

Brasilia (Escola Fazendaria), 25.V.1977, D. Gifford, 1♂ (DZUP, Coll. Gifford, ɸ), (Hmb110);

**Maranhão:**

Feira Nova do Maranhão, 13-18.IV.2011, Mielke & Casagrande, 1♀ (LMCI 184-02 ɸ Ψ^1^), 4♂ (LMCI 184-01 ɸ Ψ^1^, LMCI 184-03 Ψ^1^, LMCI 184-04 Ψ^1^, LMCI 184-05 Ψ^1^);

Imperatriz (Exc. Departamento Zoologia), 22.XII.1972, 1♀, 2♂ (DZUP ɸ), (Hmb03) (Hmb112);

Imperatriz (Exc. Departamento Zoologia), 01.VII.1974, 1♀ (DZUP ɸ), (Hmb08);

Imperatriz (Exc. Departamento Zoologia), 13.XII.1972, 1♀ (DZUP ɸ), (Hmb09);

Imperatriz (Exc. Departamento Zoologia), 15.XII.1972, 1♂ (DZUP ɸ), (Hmb106);

Imperatriz (Exc. Departamento Zoologia), 6.VII.1974, 1♂ (DZUP ɸ), (Hmb107);

Imperatriz (Exc. Departamento Zoologia), 4.VII.1974, 1♂ (DZUP ɸ), (Hmb114);

Imperatriz (Exc. Departamento Zoologia), 5.VII.1974, 1♂ (DZUP ɸ), (Hmb115);

Imperatriz (Exc. Departamento Zoologia), 15.VII.1974, 1♂ (DZUP ɸ), (Hmb116);

Imperatriz (Exc. Departamento Zoologia), 12.VII.1974, 1♂ (DZUP ɸ), (Hmb117);

Imperatriz (Exc. Departamento Zoologia), 7.VII.1974, 1♂ (DZUP ɸ), (Hmb118);

Imperatriz (Exc. Departamento Zoologia), 19.XII.1973, 2♂♂ (DZUP ɸ), (Hmb119) (Hmb 120) (Hmb 121);

**Ceara:**

Ubajara, 23.X.2011, S. Kerpel & A. Ferreira leg., 2♀ (LMCI 183-18 ɸ, LMCI 183-13 Ψ^1^), 7♂ (LMCI 183-10 ɸ Ψ^12^, LMCI 183-11 Ψ^12^, LMCI 183-12 Ψ^1^, LMCI 183-14 Ψ^12^, LMCI 183-15 Ψ^1^, LMCI 183-16 Ψ^1^)

*Heliconius melpomene nanna*

**Rio Grande do Norte:**

Natal (Parnamirin), 15.IX.1949, Moacir Alvorenga col. leg., 1♀ (DZUP, Col. D’Almeida 6664, ɸ) (Hmn01);

**Espirito Santo:**

Baixo Guandu, 11.II.1970, C. & C. T. Elias leg., 1♀ (DZUP ɸ) (Hmn10);

Colatina (Itapina), 23.XII.1967, T. Elias leg., 1♂ (DZUP ɸ) (Hmn116);

Conceição da Barra, 20.VI.1968, C. & C. T. Elias leg., 1♀ (DZUP ɸ) (Hmn05);

Conceição da Barra, 04.X.1969, C. & C. T. Elias leg., 1♀ (DZUP ɸ) (Hmn09);

Conceição da Barra, 1982, Elias leg., 1♂ (DZUP ɸ) (Hmn105);

Conceição da Barra, 4.VII.1969, C. & C. T. Elias leg., 1♂ (DZUP ɸ) (Hmn107);

Conceição da Barra, I.1986, C. & C. T. Elias leg., 1♂ (DZUP ɸ) (Hmn112);

Conceição da Barra, 10.X.1968, C. & C. T. Elias leg., 1♂ (DZUP ɸ) (Hmn119);

Conceição da Barra, 4.X.1968, C. & C. T. Elias leg., 1♂ (DZUP ɸ) (Hmn115);

Conceição da Barra, 17.XI.1969, C. & C. T. Elias leg., 1♂ (DZUP ɸ) (Hmn126);

Itapina, 23.XII.1967, T. Elias leg., 1♀ (DZUP ɸ) (Hmn02);

Jacaripe, 5.II.1967, Elias leg., 1♀ (DZUP ɸ) (Hmn04);

Linhares, R. Sooretama, 30.VII.1966, Mielke & Brown leg., 1♀, 1♂ (DZUP ɸ) (Hmn06) (Hmn109);

Linhares, R. Sooretama, 28.VII.1966, Mielke & Brown leg., 1♂ (DZUP ɸ) (Hmn108);

Linhares, XI-XII.1965, A. Maller leg., 1♀ (DZUP ɸ) (Hmn07);

Linhares, V.1981, C. Elias leg., 1♂ (DZUP ɸ) (Hmn106);

Pedro Canário, 19.VII.1977, Mielke, Moura & Elias leg., 1♂ (DZUP ɸ) (Hmn104);

Santa Teresa, 5.II.1967, C. & C. T. Elias leg., 1♀, 5♂♂ (DZUP ɸ) (Hmn08) (Hmn111) (Hmn114) (Hmn120) (Hmn121) (Hmn123);

Santa Teresa (750m), 25-29.III.1970, K. Ebert leg., 1♀ (DZUP, Coll. H. Ebert 951, ɸ) (Hmn17);

Santa Teresa, 15.III.1967, C. & C. T. Elias leg., 1♂ (DZUP ɸ) (Hmn113);

Santa Teresa, 10.IV.1966, Elias leg., 1♂ (DZUP ɸ) (Hmn118);

Santa Teresa, 11.VIII.1969, C. & C. T. Elias leg., 1♂ (DZUP ɸ) (Hmn122);

Santa Teresa, 26.IX.1968, C. & C. T. Elias leg., 1♂ (DZUP ɸ) (Hmn124);

Santa Teresa, III.1971, C. & C. T. Elias leg., 1♂ (DZUP ɸ) (Hmn129);

São Mateus, X.1985, Elias leg., 2♀♀, 1♂ (DZUP ɸ) (Hmn11) (Hmn19) (Hmn125);

Sooretama, ReBio, 28.II. -2.III.2010, GRPM et al leg., 8♂ (LMCI 105-13 Ψ^12^, LMCI 105-14 Ψ^1^, LMCI 105-15 Ψ^1^, LMCI 105-16 Ψ^1^, LMCI 105-61 Ψ^1^, LMCI 105-62 Ψ^12^, LMCI 105-63 Ψ^12^, LMCI 105-64 Ψ^1^) 1♀ (LMCI 105-65 Ψ^1^)

**Paraíba:**

VI.1953, 1♀ (DZUP, Coll. Gagarin, ɸ) (Hmn12);

24.VI.1953, J. Kesselrine leg., 1♀ (DZUP, Coll. Gagarin, ɸ) (Hmn13);

João Pessoa, Clerot leg., 1♀ (DZUP, Coll. D’Almeida 12405, ɸ) (Hmn21);

João Pessoa, 5.VII.1952, Rerrato L. Diniz col, 1♂ (DZUP,Coll. D’Almeida 11.346, ɸ) (Hmn101);

**Minas Gerais:**

Aimorés, 17.IV.1970, C. Elias leg., 1♂ (DZUP ɸ) (Hmn102);

Aimorés, 4.IV.1970, C. Elias leg., 1♂ (DZUP ɸ) (Hmn117);

**Pernambuco:**

Goiana, 7.XII.1954, H. Ebert leg, 1♀ (DZUP, Coll. H. Ebert 71, ɸ) (Hmn22);

Recife (Camaragibe, 20-80m), 27.7.1962, H. Ebert leg., 1♀ (DZUP, Coll. H. Ebert 428, ɸ) (Hmn15);

São Lourenço da Mata (Tiuma, 100m), 9.VIII.1958, H Ebert leg, 1♀ (DZUP, Coll. H. Ebert 429, ɸ) (Hmn16);

São Lourenço da Mata (Tiuma), 15.VII.1973, Mielke & Kesselring leg., ♂ (DZUP ɸ) (Hmn110);

**Bahia:**

Camacan (Serra Bonita), 17-23.II.2013, Moreira, G. R. M. leg., 1♀ (LMCI 209-26 ɸ Ψ^12^), 1♂ (LMCI 209-25 ɸ Ψ^12^);

Itamaraju (100m), 20.VII.1977, Mielke, Moura & Elias leg., 1♂ (DZUP ɸ) (Hmn103);

Itamari, 5.II.1971, Elias leg., 1♂ (DZUP ɸ) (Hmn127);

Jitaúna (Rio das Contas, 150m), 26.3.1961, Ebert, 1♀ (DZUP ɸ) (Hmn14);

Mucuri, 1.III.1971, Elias leg., 1♂ (DZUP ɸ) (Hmn128);

Prado, 5.III.1971, Elias leg., 1♂ (DZUP ɸ) (Hmn130);

São João do Paraíso (30Km N de Camacã), 22.VII.1977, Mielke, Moura & Elias leg., 1♀ (DZUP ɸ) (Hmn03);
